# Diagnosing Down-the-Drain Disposal of Unused Pharmaceuticals at a River Catchment Level: Unrecognized Sources of Environmental Contamination That Require Nontechnological Solutions

**DOI:** 10.1021/acs.est.1c01274

**Published:** 2021-08-23

**Authors:** Barbara Kasprzyk-Hordern, Kathryn Proctor, Kishore Jagadeesan, Scott Watkins, Richard Standerwick, Ruth Barden, Julie Barnett

**Affiliations:** † Department of Chemistry, University of Bath, Bath BA2 7AY, U.K.; ‡ Department of Psychology, University of Bath, Bath BA2 7AY, U.K.; § Wessex Water, Bath BA2 7WW, U.K.

**Keywords:** pharmaceuticals, disposal, WBE, wastewater-based epidemiology, at-source wastewater treatment

## Abstract

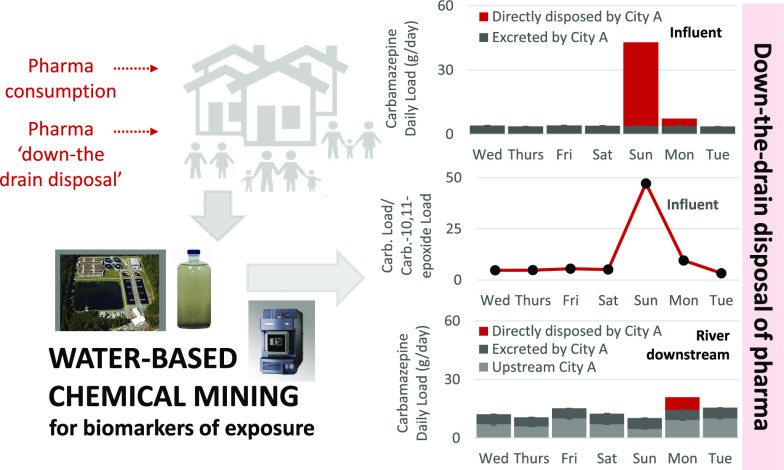

Down-the-drain disposal of pharmaceuticals remains an overlooked and unrecognized source of environmental contamination that requires nontechnological “at-source” solutions. Monitoring of 31 pharmaceuticals over 7 days in five wastewater treatment plants (WWTPs) serving five cities in South-West UK revealed down-the-drain codisposal of six pharmaceuticals to three WWTPs (carbamazepine and propranolol in city A, sildenafil in city B, and diltiazem, capecitabine, and sertraline in city D), with a one-off record codisposal of estimated 253 pills = 40 g of carbamazepine and estimated 96 pills = 4 g of propranolol in city A accounting for their 10- and 3-fold respective increases in wastewater daily loads. Direct disposal of pharmaceuticals was found to affect the efficiency of wastewater treatment with much higher pharmaceutical removal (decrease in daily load) during “down-the-drain disposal” days. This is due to lack of conjugated glucuronide metabolites that are cleaved during “consumption-only” days, with the release of a parent pharmaceutical counterbalancing its removal. Higher removal of pharmaceuticals during down-the-drain disposal days reduced pharmaceutical loads reaching receiving environment, albeit with significant levels remaining. The estimated daily loads in receiving water downstream from a discharge point accounted for 13.8 ± 3.4 and 2.1 ± 0.2 g day^–1^ of carbamazepine and propranolol, respectively, during consumption-only days and peaked at 20.9 g day^–1^ (carbamazepine) and 4.6 g day^–1^ (propranolol) during down-the-drain disposal days. Actions are needed to reduce down-the-drain disposal of pharmaceuticals. Our recent work indicated that down-the-drain disposal of pharmaceuticals doubled since the last study in 2005, which may be due to the lack of information and messaging that informs people to dispose of unused medicines at pharmacies. Media campaigns that inform the public of how to safely dispose of medicines are key to improving rates of return and reducing pharmaceutical waste in the environment. The environment is a key motivator for returning unused medicines to a pharmacy and so messaging should highlight environmental risks associated with improper disposal.

## Abstract 2

Down-the-drain disposal of pharmaceuticals remains an overlooked and unrecognized source of environmental contamination that requires nontechnological “at-source” solutions.

## 1 Introduction

Pharmaceuticals are recognized as environmental contaminants. They are released into the environment via various routes, mainly via communal discharge.^1^ There is a clear correlation between the population size in a river catchment and environmental burden resulting from pharmaceutical usage.^2,3^ There have been several papers published focused on the presence of pharmaceuticals in wastewater and receiving environment, but very little has been done to fully understand contributing sources.^2−16^ Pharmaceuticals are not regulated in water bodies; however, they are currently under scrutiny, e.g., via EU watchlists. As a result, there is not enough data on the presence of pharmaceuticals in the environment, data sets are limited to a few targets, there is limited spatial coverage at a catchment level, and there are even fewer longitudinal studies showing temporal variabilities. This does not allow for a true understanding of the scale of pharma impact on the receiving environment. One aspect that has received very little attention is accidental or intentional down-the-drain disposal of unused pharmaceuticals. A recent U.K. survey of 663 people found that 230 (35%) of them had disposed of pharmaceuticals down the sink/toilet in the past.^23^ This disposal was infrequent and may have constituted a small proportion of their leftover pharmaceuticals but could go unnoticed in the absence of high-resolution longitudinal wastewater monitoring studies or regulatory pressures, with potential for significant acute ecotoxicological effects of localized nature (those are not subject of evaluation in environmental risk assessment, ERA). This concerns both the performance of wastewater treatment plants, as wastewater treatment is biological in nature, and the receiving aquatic environment. A number of studies have demonstrated that pharmaceutical concentrations in water have influenced a range of behaviors in fish that are important for fitness, food-web properties, and ecosystem functioning.^17^ We have previously reported direct one-off disposal of 915 capsules of fluoxetine^18^ in our earlier study. We have assumed that it is unlikely to be at the patient level and postulated that direct disposal was from a facility that handles larger quantities of the drug (e.g., a pharmacy). In contrast, a study of university students’ disposal patterns did not indicate down-the-drain disposal as an important route of pharmaceuticals reaching wastewater over a 10 day long study in a population of 30 000 served by one WWTP.^19^

**Figure 1:**
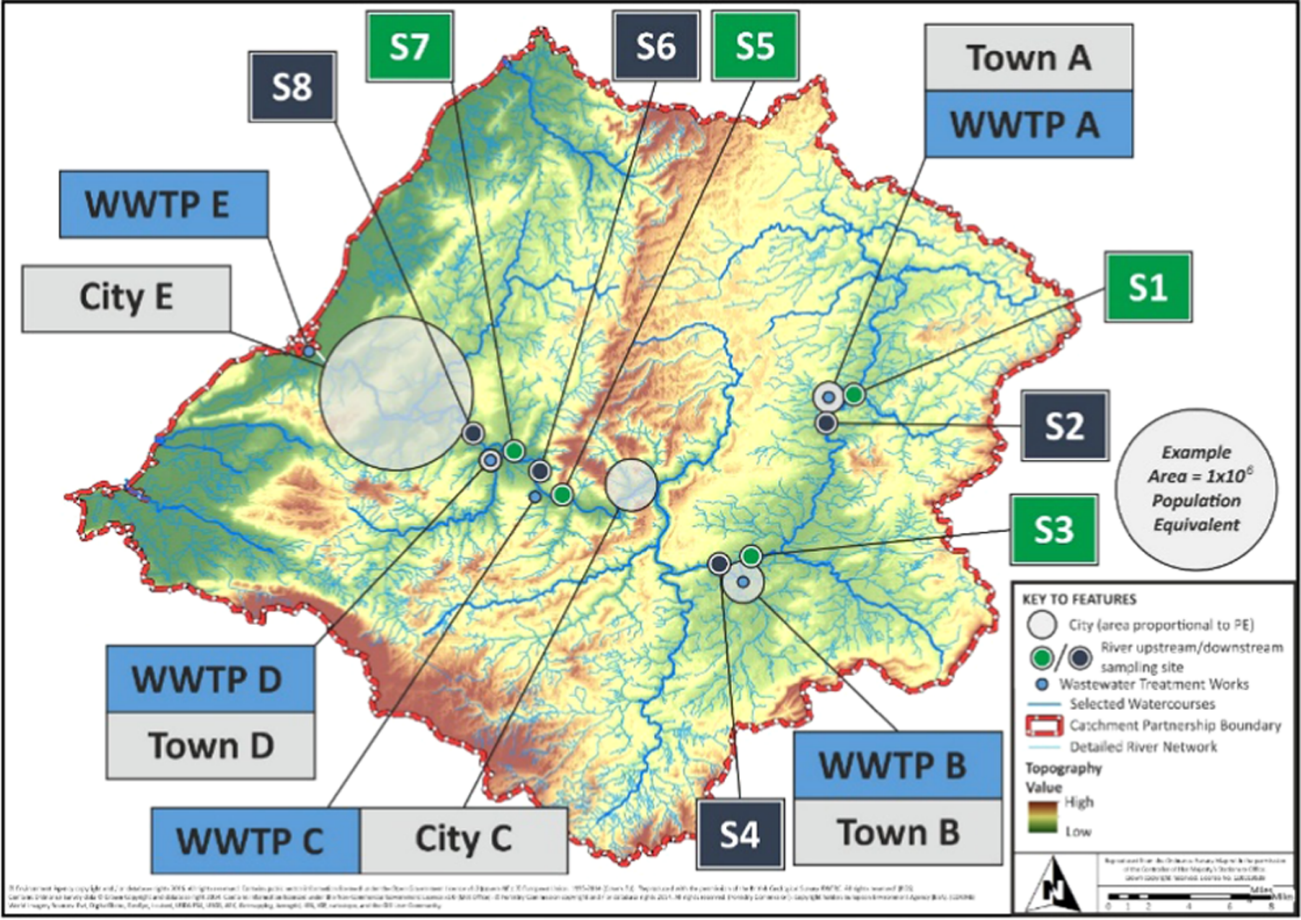
Site information of studied WWTPs and corresponding river locations (note: towns A, B, and D are called cities A, B, and C, respectively, in the text for simplicity reasons).

This paper focuses on understanding the frequency of down-the-drain disposal of pharmaceuticals in five contrasting towns/cities served by five major WWTPs (Figure 1, sites A–E) contributing to one river catchment in the South-West UK and covering an area of approximately 2000 km^2^ and the population of ∼1.5 million (this constitutes >75% of the overall population in the catchment). It also aims to assess environmental impacts resulting from down-the-drain disposal of unused pharmaceuticals.

## 2 Materials and Methods

### 2.1 Reagents and Analytical Standards

Several groups of pharmaceuticals were studied (Table 1). Water was purified using a Milli-Q purification system from Millipore (Nottingham, U.K.). Methanol, formic acid (>95%), HCl (concentrated), 1 M NaOH, 1 M NH_4_OH, NH_4_F, and 2-propanol were purchased from Sigma-Aldrich (U.K.) and Fisher (U.K.). All solvents used were of high-performance liquid chromatography (HPLC) grade or higher. All glassware was deactivated using a 5% (v/v) dimethyldichlorosilane (DMDCS) in toluene (Sigma-Aldrich, U.K.) to prevent losses from analyte sorption according to the procedure described elsewhere.^20^

**Table 1:** Pharmaceuticals and Their Metabolites Targeted in This Study

class of analyte	analyte	metabolite
antibiotics and antibacterial agents	sulfasalazine	
	clarithromycin	
	azithromycin	
	trimethoprim	
	sulfamethoxazole	
hypertension	valsartan	
	irbesartan	
	lisinopril	
NSAIDs	ibuprofen	
	naproxen	
	diclofenac	
lipid regulator	bezafibrate	
	atorvastatin	
diabetes	metformin	
	gliclazide	
	sitagliptin	
β-blocker	atenolol	
	metoprolol	
	propranolol	
	bisoprolol	
opioids	buprenorphine	
antidepressants	venlafaxine	desmethylvenlafaxine
	fluoxetine	norfluoxetine
	mirtazapine	
	citalopram	desmethylcitalopram
	amitriptyline	nortriptyline
	sertraline	norsertraline
antiepileptic	carbamazepine	carbamazepine-10,11-epoxide
calcium-channel blocker	diltiazem	
hypnotic	temazepam	
	oxazepam	
other	sildenafil	
	capecitabine	

### 2.2 Sample Collection

Untreated wastewater samples were collected at wastewater treatment plants (WWTPs) after physical screening (course screens) for 7 consecutive days from Wednesday to Tuesday between June and October 2015 from five major WWTPs in South-West England (Figure 1, sites A–E, 1 week per site) contributing to one river catchment, the population of ∼1.5 million (>75% of the overall population in the catchment). Further information on the catchment can be found elsewhere.^2^

Influent was collected as volume proportional 24 h composites with average subsample collection frequencies of approximately 15 min, and effluent wastewater was collected as time proportional 24 h composites with subsamples every 15 min, using an ISCO 3700 autosampler packed with ice to maintain 4 °C to limit biological activity (see WWTP A–E in Figure 1). River water samples were collected as grab samples on the same days as wastewater samples (see S1–S8 in Figure 1). All samples were transported on ice to the laboratory, spiked with the internal standards, and stored at −18 °C until sample preparation and analysis could take place.

### 2.3 Sample Preparation and Analysis

Full pharmaceutical mass balance in wastewater was calculated based on concentrations of pharmaceuticals in both liquid and solid phase fractions. Solid phase extraction (SPE) was used for the extraction of pharmaceuticals from the liquid phase. Microwave-assisted extraction (MAE) followed by SPE was used for the extraction of pharmaceuticals from the solid phase. UHPLC-QqQ (ultraperformance liquid chromatography and tandem triple quadrupole mass spectrometry) method was utilized for targeted analysis of pharmaceuticals and their metabolites. Detailed description of the method and full method performance parameters can be found elsewhere.^20^

Briefly, liquid samples (50 mL) were filtered using a GF/F 0.75 μm glass microfiber filter (Fisher Scientific, U.K.), adjusted to pH 7.5 ± 0.1 and spiked with 50 ng of internal standards’ solution (50 μL of 1 μg mL^–1^ in MeOH). Solid phase extraction (SPE) was performed using Oasis HLB sorbents (Waters, U.K.), which were conditioned using 2 mL of MeOH followed by 2 mL of H_2_O at 1 mL min^–1^. Samples were then loaded at 5 mL min^–1^ and dried under vacuum. Elution was undertaken using 4 mL of MeOH at a rate of 1 mL min^–1^. Methanolic extracts were subsequently dried under nitrogen using a TurboVap evaporator (Caliper, U.K., 40 °C, *N*^2^, <5 psi). Dried extracts were reconstituted in 500 μL of 80:20 H_2_O/MeOH and then analyzed with UHPLC-QqQ.

Suspended particulate matter (SPM) obtained from GF/F filters was freeze-dried, and 0.25 g samples were spiked with 50 ng of internal standard solution (50 μL of 1 μg mL^–1^ in MeOH). MAE was used as described elsewhere.^20^ Briefly, samples in 25 mL of 50:50 MeOH/H_2_O (pH 2) were heated at 110 °C using an 800 W MARS 6 microwave (CEM, U.K.). MAE extracts were then adjusted to <5% of MeOH using H_2_O (pH 2), passed through preconditioned Oasis MCX SPE cartridges (Waters, U.K.), and eluted in two fractions: the acidic pharmaceuticals with 2 mL of 0.6% HCOOH in MeOH followed by the basic pharmaceuticals with 3 mL of 7% NH_4_OH in MeOH. Once dried, the extracts were reconstituted in 500 μL of 80:20 H_2_O/MeOH and filtered using pre-LCMS 0.2 μm poly(tetrafluoroethylene) (PTFE) filters (Whatman, Puradisc). SPM was analyzed only in wastewater influent due to difficulties in obtaining SPM from effluent and river water.

Extracted analytes were separated on a BEH C18 column (150 × 1.0 mm^2^, 1.7 μm particle size) (Waters, Manchester, U.K.) with a 0.2 μm, 2.1 mm in-line column filter using a Waters Acquity UPLC system (Waters, Manchester, U.K.) and quantified with a Xevo TQD Triple Quadrupole Mass Spectrometer (Waters, Manchester, U.K.) equipped with an electrospray ionization source. Analysis was performed in both ESI+ and ESI– with a capillary voltage of 3.20 kV, a desolvation temperature of 400 °C, and a source temperature of 150 °C. Nitrogen was used as the nebulizing and desolvation gas and argon as the collision gas. The cone gas flow was 100 L h^–1^, and the desolvation gas flow was 550 L h^–1^. See Figure S1, Table S1, and paper by Proctor et al.^20^ for further details regarding the method and its performance parameters.

### 2.4 Prescription Data

Consumption of prescribed pharmaceuticals for the WWTP catchments involved in this study was calculated using an R package, PrAna, developed in our research group (http://pranaviz.bath.ac.uk). PrAna package uses England’s national level monthly prescription data published by NHS Digital (https://digital.nhs.uk/) to aggregate, normalize, and map each prescribed pharmaceutical to its corresponding prescribing general practice (GP) surgeries and postcode for the period 2015–2019. PrAna package also features PrAnaViz, a web-based interactive tool to visualize and analyze PrAna-generated data set in real time. PrAnaViz facilitates wider use with spatiotemporal and long-term trends. WWTP catchment maps were used to identify GP surgeries inside each catchment region to collect their information. For this study, we have extracted the identified GP surgery prescriptions of the pharmaceutical drugs of different pharmacological groups including antidepressants, antidiabetics, antimicrobial, cardiovascular agents, and antianxiety/antidepressants. The data were normalized to the quantity (kg month^–1^) of individual pharmaceutical compounds prescribed in each postcode inside the catchment zone. The average amount prescribed each day for that month (mg day^–1^) was calculated from the monthly consumption quantity. We have used prescription data from June 2015 to October 2015, mirroring the sampling months for each WWTP site.

### 2.5 Calculations

Daily mass loads of pharmaceuticals (mg day^–1^) were calculated by multiplying the total pharma concentrations (mg L^–1^) in a 24 h composite raw wastewater sample by daily wastewater flow rates (L day^–1^). Total pharma concentrations in raw wastewater were calculated after taking into account both liquid and SPM fractions
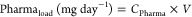
where *C*_Pharma_ is the total concentration of pharma (mg L^–1^) in influent wastewater (both liquid and SPE phase), and *V* is the volume of wastewater received by the WWTP per day (L day^–1^).

Mass loads (mg day^–1^) were then normalized to the number of people served by each WWTP (mg day^–1^ 1000 inhabitants^–1^) to give population normalized mass loads (PNDLs) to compare results between different WWTPs
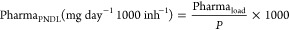
where *P* is the population size served by WWTPs.

Estimated number of pills disposed of down-the-drain was calculated using the following formula
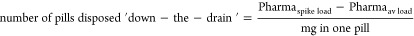
where pharma_spike load_ is the pharma daily spike load (mg day^–1^) and pharma_av load_ is the pharma average daily “typical” load (mg day^–1^); mg in one pill is a weighted average calculated from the above-mentioned prescription data (containing number of items prescribed, including number of pills in each item prescribed and the quantity of an active substance in each pill) from June 2015 to October 2015, mirroring the sampling months for the each WWTP site:
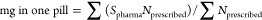
where the strength of pharma (*S*_pharma_, in mg) is the quantity of active pharmaceutical in each item prescribed within each WWTP catchment area (measured during every month of sample collection) and the number of items prescribed (*N*_prescribed_) is the number of pharmaceutical items prescribed within each WWTP catchment area (measured during every month of sample collection).

Please note that we decided not to use the defined daily dose (DDD) as it is only the assumed average maintenance dose per day for some drugs used in adults. Our calculations focused on the high-resolution pharma prescription data set per postcode and per month in the catchment area to provide the best estimates possible.

Estimated number of pills consumed was calculated using the following formula
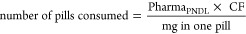
where CF is the correction factor. It was calculated using the following formula
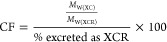
where *M*_W(XC)_ is the molecular weight of XC (pharma), and *M*_W(XCR)_ is the molecular weight of XCR (metabolite of pharma).

CF used for carbamazepine was 7.1 (with carbamazepine used as a drug target marker) (see the detailed discussion in Kasprzyk-Hordern et al.^21^).

RQ (a risk quotient) for carbamazepine was calculated using the following formula
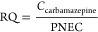
where PNEC is the predicted no-effect concentration (ng L^–1^) and *C*_Carbamazepine_ is the estimated carbamazepine concentration (ng L^–1^) calculated by dividing the estimated daily load of carbamazepine by the daily flow of river water at sample collection point (see Proctor et al. 2021 for further information and SI data sets for pharmaceutical loads and flows^2^).

## 3 Results and Discussion

Several groups of pharmaceuticals (>30 pharmaceuticals and their metabolites) were investigated in this unique large-scale study focused on five towns and cities in the Avon river catchment, South-West England, (Figure 1), via wastewater-based epidemiology. These are NSAIDs, antidiabetics, cardiovascular agents, antidepressants, and antibiotics.

### 3.1 Pharmaceuticals in Raw Wastewater

#### 3.1.1 Evidence of Direct Disposal

**Figure 2:**
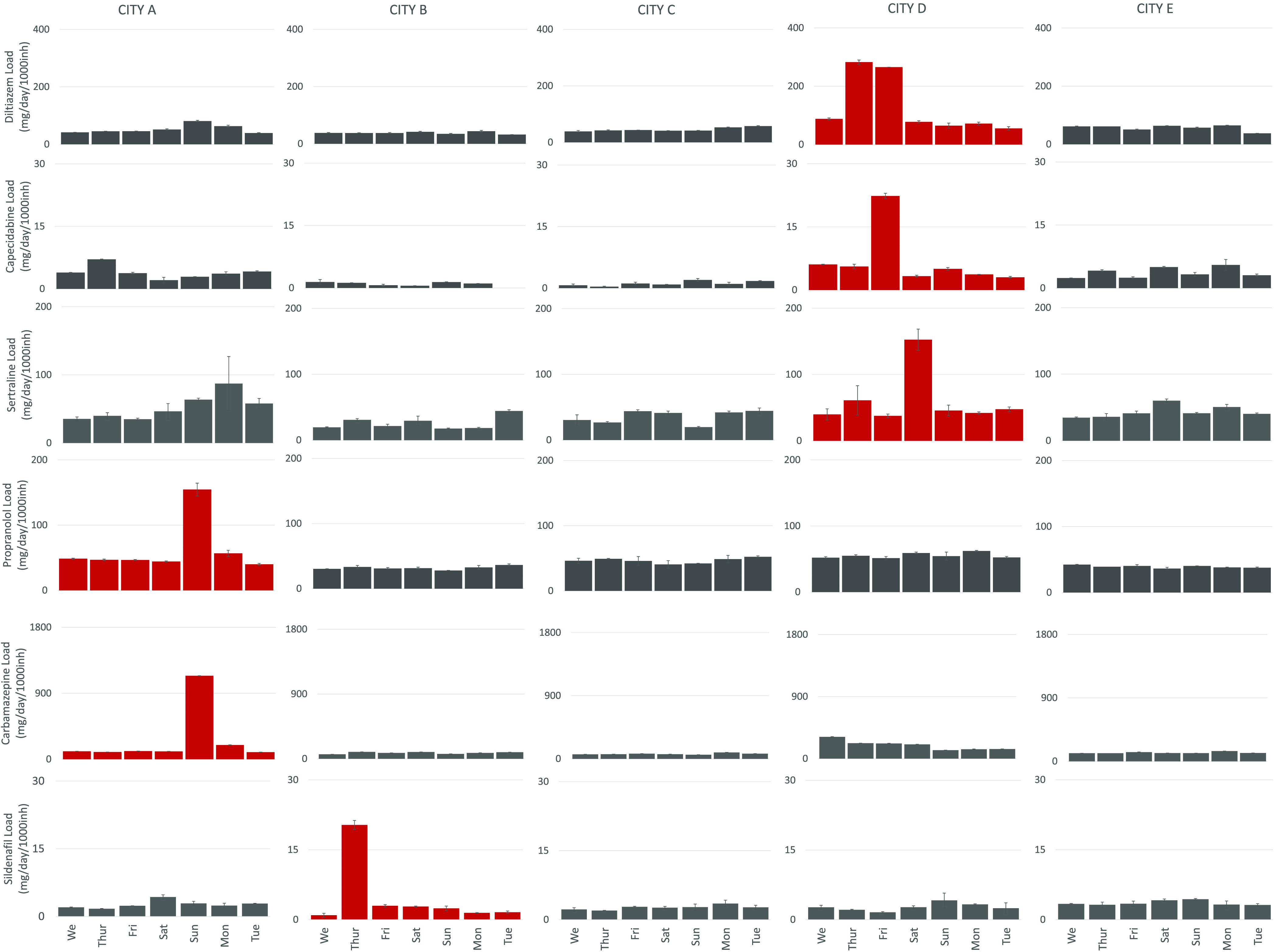
Population normalized daily loads of pharmaceuticals with evidence of direct disposal.

Thirty-one pharmaceuticals were monitored over 7 days in five WWTPs serving five cities. On six occasions, PNDLs of pharmaceuticals were found to deviate from weekly trends in WWTPs D, A, and B serving towns with approximately 18 000, 38 000, and 68 000 people. These are carbamazepine and propranolol in city A, sildenafil in city B, and diltiazem, capecitabine, and sertraline in city D. No deviation from weekly baseline was observed in larger cities C and E with 110 000 and 867 000 inhabitants (Figure 2 and Table S2). This can be explained by the much larger quantity of consumed pharmaceuticals discharged to the sewerage system that masks any deviation from the trendline resulting from direct disposal. It is important to remember that each town/city was monitored only for 1 week. Due to the assumed random nature of pharma disposal, one cannot provide definite answers regarding why cities A, B, and D had direct disposal recorded and why cities C and E had not.

Estimated 275 pills of carbamazepine accounting for >40 g and estimated 96 pills accounting for 4 g of propranolol were disposed (likely in one dumping event) down-the-drain in city A on Sun/Mon (Table 2). This added an additional 40 and 4 g of carbamazepine and propranolol, respectively, to the average daily levels of these pharmaceuticals in wastewater: 3.9 and 1.8 g, respectively. This is a significant 10- and 2-fold increase in daily loads of carbamazepine and propranolol, respectively, reaching wastewater treatment works.

**Table 2:** Estimated Number of Pills Disposed of Down-the-Drain

pharmaceutical		mg in one pill^a^	estimated no. of pills disposed of “down-the-drain”	mg day^–1^ disposed of down-the-drain^c^	average daily load (mg day^–1^) of pharma in wastewater resulting from consumption (quantity excreted)^d^	% increase in daily pharma load due to “down-the-drain disposal” (%)^e^
carbamazepine	city A	154	253 (Sun) 22 (Mon)	39 013 (Sun) 3411 (Mon)	3934.0 ± 189.4	992
						87
propranolol	city A	42	96 (Mon)	4050.6 (Mon)	1778.4 ± 191.0	278
sildenafil	city B	77	16 (Thur)	1252 (Thur)	139.3 ± 51.4	899
sertraline	city D	71	27.5 (Sat)	1951.8 (Sat)	834.2 ± 140.0	234
diltiazem	city D	146	26.4 (Thurs) 24.3 (Fri)	3856.2 (Thur) 3544.6 (Fri)	1306.1 ± 203.2	295
						271
capecitabine	city D	b	- (Fri)	339.6 (Fri)	81.3 ± 21.4	298

a Weighted average calculated from NHS prescription data: , where *S*_pharma_ is the strength of the pharma (in mg) and *N*_prescribed_ is the number of items prescribed.

b Not prescribed; mg in one pill could not be calculated.

c mg day^–1^ disposed of down-the-drain = total pharma load in wastewater influent during disposal day (mg day^–1^) – average daily load in wastewater influent on nondisposal days (mg day^–1^).

d Average daily load (mg day^–1^) of pharma in wastewater resulting from consumption (quantity excreted) = total pharma load in wastewater influent during disposal day (mg day^–1^) – pharma load in wastewater influent resulting from disposal (mg day^–1^).

e % increase in daily pharma load due to down-the-drain disposal = mg day^–1^ disposed of down-the-drain × 100/mg day^–1^ disposed of down-the-drain.

City B has seen an increase in 1.3 g of sildenafil on Thursday, which equals estimated 16 pills disposed of down-the-drain and indicates a 9-fold increase in daily levels. Estimated 50 pills of diltiazem (7.3 g) and estimated 28 pills of sertraline (2 g) were disposed of down-the-drain during two different dumping events in city D, which accounted for 2- to 3-fold increase in daily loads.

We have taken a conservative approach in estimating direct disposal. Our calculations were applied to drugs for noncommunicable diseases that do not show interday (weekday–weekend) changes in usage patterns (with the exception of erectile dysfunction sildenafil showing a small increase in usage over the weekend); hence, we assumed it is appropriate to use the weekly average compound-dependent trendline.

#### 3.1.2 Role of Metabolites in Confirmation of Down-the-Drain Disposal of Pharmaceuticals

**Figure 3:**
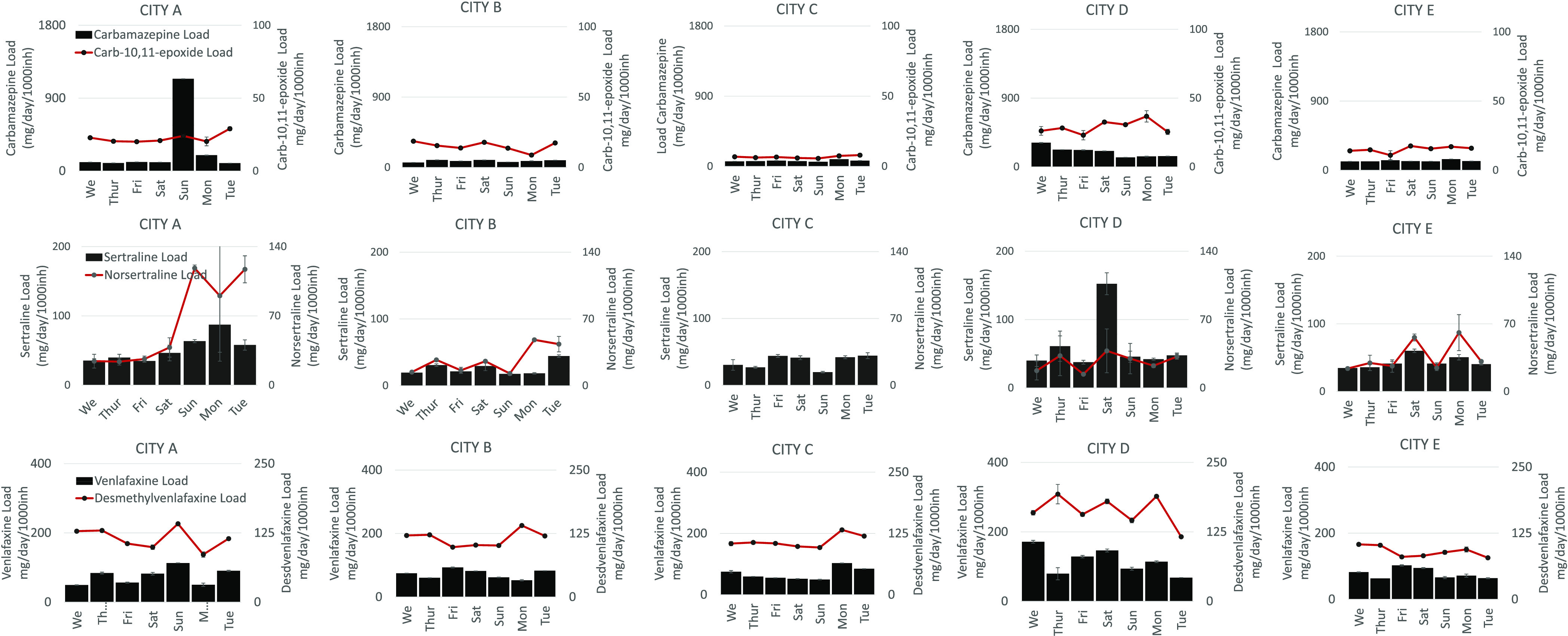
Population normalized daily loads of pharmaceuticals and their metabolites.

**Figure 4:**
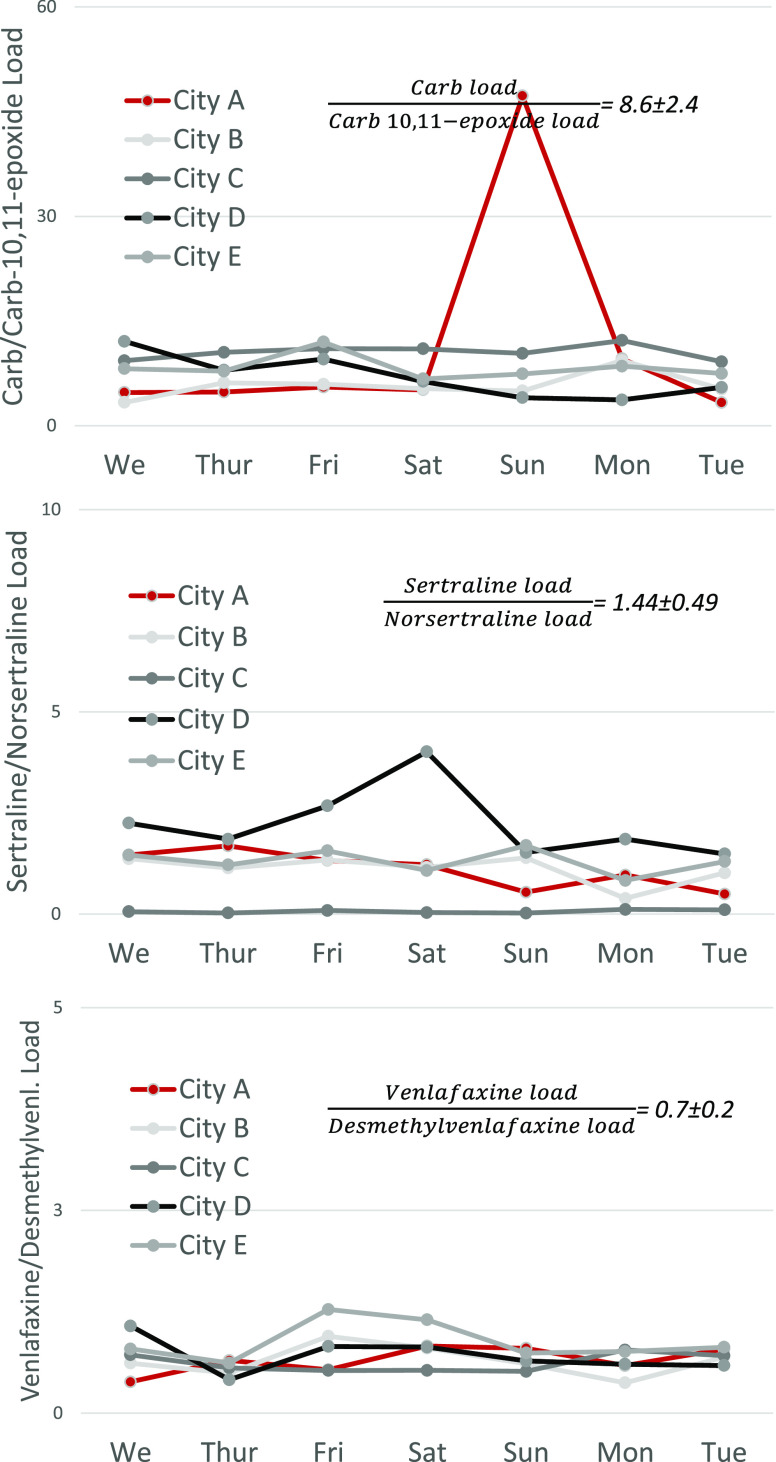
Pharma/pharma metabolite ratios.

While deviation of pharmaceutical levels from the consumption baseline is a good indication of down-the-drain disposal, an understanding of parent compound/metabolite ratio baseline provides further confirmation of a direct disposal event occurring. As seen in Figures 3 and 4, all pharmaceuticals reveal relatively stable parent compound/metabolite ratios with the exception of carbamazepine’s spike on Sunday in WWTP A and sertraline’s spike on Saturday in WWTP D. Both pharmaceuticals experienced increased parent compound loads on these 2 days despite unchanged and constant metabolite daily loads. This evidences direct disposal. Interestingly, sertraline spike on Sun–Tue in City A was linked with higher consumption (and not direct disposal) as the loads of both sertraline and its metabolite norsertraline increased with unchanged parent compound/metabolite ratio.

An understanding of the parent compound/metabolite ratio baseline is also critical in disregarding “false-positive” cases of direct disposal. For example, knowledge of the parent compound/metabolite ratio disregarded higher venlafaxine levels on Sun/city A and Mon/city C as down-the-drain disposal cases. This is because both an increase of parent compound and metabolite daily load indicated an increase in consumption of the drug. Interestingly, in city D, loads of venlafaxine remained variable despite stable desmethylvenlafaxine loads, which might indicate several cases of relatively small events of venlafaxine down-the-drain disposal. It is important to mention that no metabolites were analyzed for sildenafil, capecitabine, and propranolol. Therefore, suspected direct disposal of these drugs remains unconfirmed.

### 3.2 Impact of Down-the-Drain Disposal of Pharmaceuticals on Receiving Environment: Carbamazepine Example

**Figure 5:**
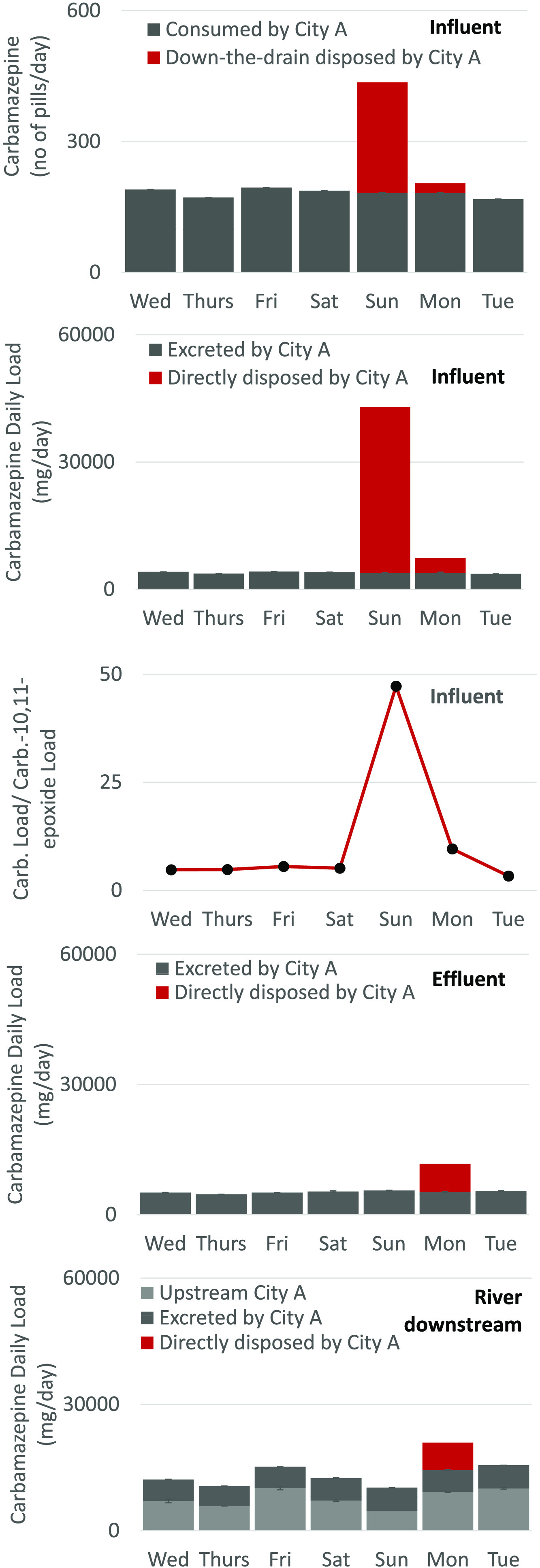
Estimated number of pills disposed of down-the-drain vs estimated number of pills consumed in cities with evidence for direct disposal.

The average daily consumption of carbamazepine pills is population-size-driven and varied from 165 per day in city D to 4931 in city E. Down-the-drain disposal of carbamazepine was observed in city A on Sunday and Monday (Figure 2). This was confirmed by the increased carbamazepine/carbamazepine-10,11-epoxide ratio (Figure 4). Based on the weighted average of 154 mg per carbamazepine per pill, 253 pills followed by 22 pills were estimated to be disposed of down-the-drain on Sunday and Monday. Therefore, the quantity of estimated pills disposed of on the 2 days (275 pills) was higher than actual community-wide daily consumption (182 ± 9) (Figure 5).

**Figure 6:**
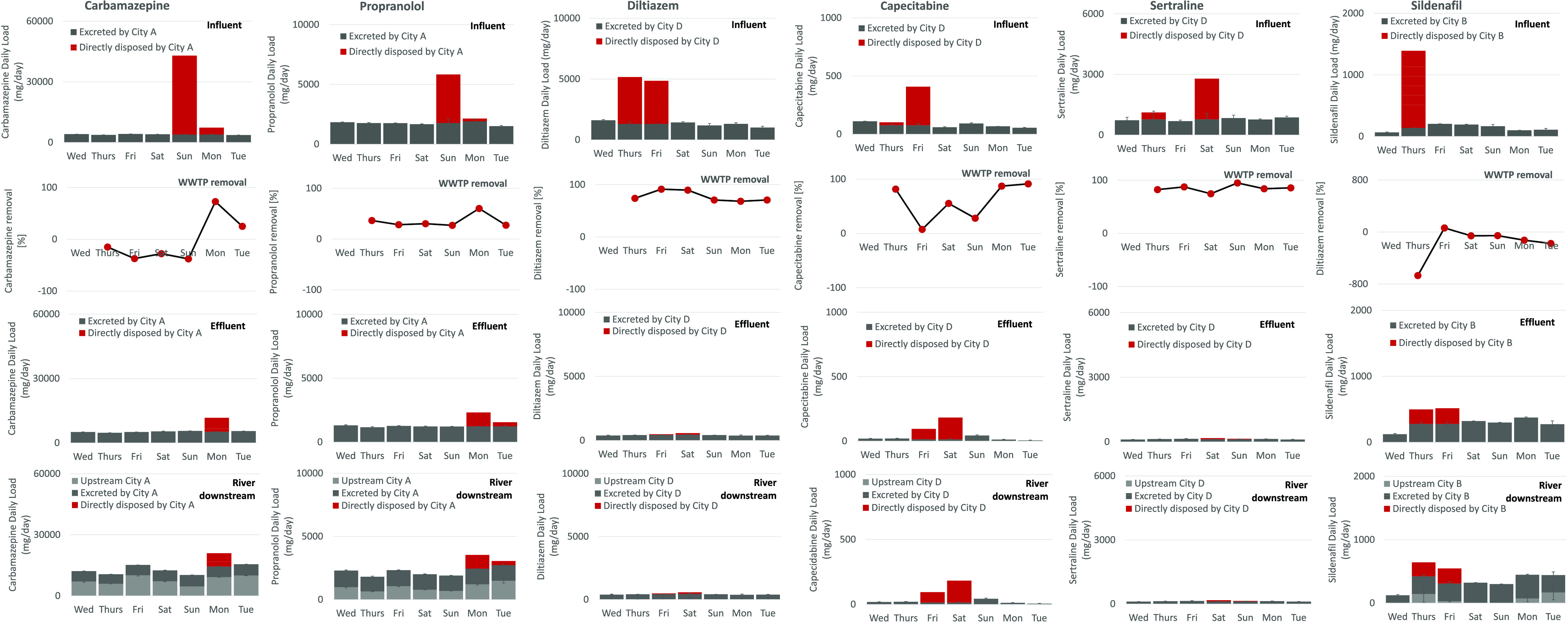
Down-the-drain codisposal of pharmaceuticals and resulting environmental burden.

On average, 5.2 ± 0.3 g day^–1^ of carbamazepine was found in wastewater effluent, which, when compared to the average wastewater influent daily loads accounting for 3.9 ± 0.2 g day^–1^, indicates that no carbamazepine was removed during wastewater treatment. Indeed, as shown in Figure 6, during carbamazepine “consumption days”, −29.5% removal was observed. Potential cleavage of phase II metabolites might have taken place, increasing carbamazepine loads in wastewater effluent leading to the negative removal. Interestingly, while 39 g of down-the-drain disposed carbamazepine was found in wastewater influent on Sunday, only 6.4 g was quantified in wastewater effluent on Monday (time lag is due to the hydraulic retention time at WWTP A accounting for up to 46 h). This indicates an increase in carbamazepine’s removal from the treatment process during carbamazepine “disposal days” (up to 72%). This is an interesting outcome confirming that wastewater treatment is effective in the removal of carbamazepine. However, in the presence of phase two conjugated metabolites, cleavage of free carbamazepine counterbalances its removal.

Daily loads of carbamazepine in river water, upstream from a discharge point, were estimated based on grab sampling, with average daily loads throughout the sampling week accounting for 7.7 ± 1.9 g day^–1^ (Figure 1, point S1). With additional load discharged by WWTP A (5.2 ± 0.3 g day^–1^), the estimated daily carbamazepine loads in the river were 13 g and peaked on Monday at 21 g, the day after carbamazepine’s down-the-drain disposal.

Considering the potential effects on the environment, the estimated carbamazepine concentration was compared with the PNEC of carbamazepine, 0.05 μg L^–1^.^36^ It showed high average risk on “consumption-only” days with an RQ value of 2.7 ± 0.4 on average (136 ± 17.9 ng L^–1^), whereas on Sunday, during the disposal event, the RQ was 3.0. There was therefore no significant fluctuation in risk for the metabolite, with average RQ values of 0.034 ± 0.010 on consumption days and 0.030 ± 0.013 on disposal days.

### 3.3 Impact of Down-the-Drain Codisposal of Pharmaceuticals on the Measured Efficiency of Wastewater Treatment and Resulting Burden on the Receiving Environment

As discussed above, codisposal of pharmaceuticals was observed in cities A and D (Figure 2). In city A, both estimated 353 pills of carbamazepine and 96 pills of propranolol were disposed of on Sunday. This accounted for an additional 39 g of carbamazepine and 4 g of propranolol entering wastewater. Interestingly, as opposed to carbamazepine that observed an increase in daily loads in WWTP effluent (average daily removal −29.5 ± 9.1% during consumption-only days and overall, −3.4 ± 40.2%), propranolol was removed from wastewater, with the average daily percentage removal accounting for 30.5 ± 11.7%. Although, as in the case of carbamazepine, the measured propranolol’s removal appeared higher during disposal days (up to 60.2%) vs consumption days (30.6 ± 3.6%) (Figure 6).

In city D, codisposal of three pharmaceuticals was observed: diltiazem (estimated 50.7 pills), capecitabine (not prescribed in the region in primary care according to official statistics, with likely disposal due to prescription in secondary care), and sertraline (estimated 27.5 pills), albeit sertraline seems to have been disposed of up to 1 day later. This accounted for an additional 7.3 g of diltiazem, 0.3 g of capecitabine, and 2 g of sertraline entering wastewater. As opposed to carbamazepine, wastewater treatment was effective in the removal of diltiazem (77.4 ± 9.4% during consumption days, with up to 91% during disposal days), capecitabine (77.1 ± 10.9%), and sertraline (84.8 ± 6.1% during consumption days, with up to 95% removal during disposal days) leading to lower environmental burden (Figure 6). Similarly, singular disposal of estimated 16 pills of sildenafil (1.3 g) in city B leads to 60.3% removal when compared to consumption-only days (−216.7 ± 230.7%). It is important to note that the increased measured efficiency of pharmaceuticals’ removal during disposal days is unlikely linked with the increased performance of treatment but a result of lower percentage of pharmaceuticals cleaved due to glucuronide deconjugation when compared with large quantities of “additional” directly disposed (unmetabolized) pharmaceutical load. Interestingly, capecitabine is known not to metabolize in humans via glucuronidation^35^ and no measured increase in the removal of capecitabine was observed. Further work is needed to understand this phenomenon.

High performance of wastewater treatment processes reduced loads reaching receiving environment, albeit with significant levels remaining. Daily loads of river water upstream from a discharge point accounted for 7.7 ± 1.9 g day^–1^ (carbamazepine), 1.0 ± 0.3 g day^–1^ (propranolol), and 0.1 ± 0.1 g day^–1^ (sildenafil). No diltiazem, capecitabine, and sertraline were quantified in upstream river water. With additional load discharged, estimated daily loads in river water downstream from a discharge point accounted for 13.8 ± 3.4 g day^–1^ (carbamazepine), 2.1 ± 0.2 g day^–1^ (propranolol), 420.7 ± 22.8 mg day^–1^ (diltiazem), 22.1 ± 10.8 mg day^–1^ (capecitabine), 123.8 ± 10.6 mg day^–1^ (sertraline), and 295.2 ± 96.8 mg day^–1^ (sildenafil) during consumption days and spiked at 20.9 g day^–1^ (carbamazepine), 4.6 g day^–1^ (propranolol), 571 mg day^–1^ (diltiazem), 182.6 mg day^–1^ (capecitabine), 175.2 mg day^–1^ (sertraline), and 642.2 mg day^–1^ (sildenafil) during disposal days.

This study reported spikes in carbamazepine, propranolol, sildenafil, sertraline, diltiazem, and capecitabine throughout towns and cities contributing to one river catchment in the South-West UK. High performance of wastewater treatment processes reduced loads reaching receiving environment. However, many of these compounds have been demonstrated in previous work to affect the behavior and/or biological make-up of aquatic life.^22−29^ Hence, actions are needed to reduce down-the-drain disposal of pharmaceuticals.

### 3.4 Raising Awareness of Correct Disposal

We identified spikes in carbamazepine and sertraline independent of metabolic load, which indicated direct disposal into the water system. Household disposal of medicines is a global issue,^30^ with differences in disposal behavior related to policy, education, and culture.^31^ A recent U.K. study suggests that disposal down toilets and sinks may have doubled since the last study in 2005,^37^ which may be due to the lack of information and messaging that informs people to dispose of unused medicines at pharmacies. The same study reported that 42% of people were unaware that they could return unused medicines to a pharmacy and only 27% could recall receiving information on correct disposal. Media campaigns that inform the public of how to safely dispose of medicines are key to improving rates of return and reducing pharmaceutical waste in the environment.^31−34^ The environment is a key motivator for returning unused medicines to a pharmacy^31^ and so messaging should highlight environmental risks associated with improper disposal.^34^ Clear disposal labeling on medicine packets would also increase awareness and may be particularly relevant to liquid medicines, which in a recent U.K. study were found to be 5 times more likely to be flushed than a solid.^37^ While the United Kingdom is ahead of many countries in its rates of pharmacy return,^30^ it is far behind countries like Sweden who have a formalized and sustained system for disposal of unused medicines in place.^34^

## Supporting Information Available

SPE/MAE-UHPLC-QqQ, schematic overview; daily loads of studied pharmaceuticals in wastewater influent; and daily loads of studied pharmaceuticals (with suspected direct disposal) in wastewater influent and effluent (PDF)
