# Supplementary material for: Diagnosing Down-the-Drain Disposal of Unused Pharmaceuticals at a River Catchment Level: Unrecognized Sources of Environmental Contamination That Require Nontechnological Solutions
Source: Environ Sci Technol. 2021 Aug 23;55(17):11657–66. doi: 10.1021/acs.est.1c01274 (PMC8735766; doi:10.1021/acs.est.1c01274)
Supplement: Supplementary file 1 — es1c01274_si_001.pdf [file es1c01274_si_001.pdf]

Supplementary Material

Diagnosing down-the drain disposal of unused pharmaceuticals at a river catchment level: unrecognised sources of environmental contamination that require non-technological solutions

Barbara Kasprzyk-Hordern[a1], Kathryn Proctor[a], Kishore Jagadeesan[a], Scott Watkins[b], Richard Standerwick[c], Ruth Barden[a,c], Julie Barnett[b]

[a]Department of Chemistry, University of Bath, Bath BA2 7AY, UK
[b]Department of Psychology, University of Bath, BA2 7AY, UK
[c]Wessex Water, Bath BA2 7WW, UK

Table S1 SPE/MAE-UHPLC-QqQ - method performance (taken from Proctor et al., 2019 [1].

**Class of Analyte**	**Analyte**	Internal Standard	Inter-day instrumental performance		Influent (ng L^{-1})		SPM (ng g^{-1})	
			Prec. (%)	Acc. (%)	***MDL***	***MQL***	***MDL***	***MQL***
Antibiotics and Antibacterial	Sulfasalazine	Naproxen-d3	2.4	104.7	12.6	41.4	-	-
	Clarithromycin	Methadone-d9	2.4	101.8	0.34	1.69	-	-
	Azithromycin	Verapamil-d7	1.5	102.0	0.14	0.45	0.03	0.10
	Trimethoprim	Methamphetamine-d5	2.2	99.5	0.73	2.41	0.01	0.03
	Sulfamethoxazole	Benzoylecgonine-d8	2.4	96.0	0.72	2.38	0.02	0.08
Hypertension	Valsartan	Naproxen-d3	3.5	118.6	7.24	23.9	-	-
	Irbesartan	Bisphenol A-d16	4.1	98.3	2.50	12.5	-	-
	Lisinopril	Amphetamine-d5	7.2	95.2	3.25	32.5	0.04	0.43
NSAIDs	Ibuprofen[a]	Ibuprofen-d3	2.3	94.2	0.19	0.93	0.005	0.02
	Naproxen	Naproxen-d3	2.5	98.3	6.29	31.5	0.05	0.25
	Diclofenac[a]	Naproxen-d3	4.5	91.8	0.67	2.22	0.02	0.06
Lipid regulator	Bezafibrate	Bezafibrate-d6	2.8	97.9	0.64	2.11	0.02	0.05
	Atorvastatin	Naproxen-d3	3.5	100.9	0.17	0.85	-	-
Diabetes	Metformin	Metformin-d6	1.3	97.0	457.0*	1509*	-	-
	Gliclazide	Quetiapine-d8	2.8	95.3	0.22	1.09	-	-
	Sitagliptin	Verapamil-d7	3.0	110.3	0.06	0.22	0.004	0.01
Beta-blocker	Atenolol	Atenolol-d7	2.3	96.8	0.71	2.35	0.01	0.05
	Metoprolol	Metoprolol-d7	2.0	96.1	0.28	1.40	0.01	0.03
	Propranolol	Propranolol-d7	1.0	106.2	0.68	2.25	0.01	0.04
	Bisoprolol	Sildenafil-d8	2.0	96.0	0.003	0.01	0.0001	0.0005
Various	Buprenorphine	Amitriptyline-d3	11.5	88.2	0.18	0.61	0.02	0.07
Antidepressants and metabolites	Venlafaxine	Metoprolol-d7	1.7	90.5	0.37	1.83	0.01	0.03
	Desmethylvenlafaxine	Metoprolol-d7	2.1	102.3	0.85	2.79	0.01	0.05
	Fluoxetine	Fluoxetine-d5	1.8	98.3	0.50	2.52	0.005	0.02
	Norfluoxetine[a]	Fluoxetine-d5	3.1	103.1	0.42	2.12	0.004	0.02
	Mirtazapine	Mirtazapine-d3	2.7	97.6	0.39	1.94	0.01	0.03
	Citalopram	Citalopram-d6	2.6	101.8	1.24	12.4	0.02	0.24
	Desmethylcitalopram	Citalopram-d6	3.0	103.4	0.31	1.54	0.01	0.03
	Amitriptyline	Amitriptyline-d3	2.4	96.8	0.30	1.02	0.02	0.07
	Nortriptyline	Nortriptyline-d3	3.1	92.9	0.61	2.03	0.03	0.10
	Norsertraline	Norsertraline-d4	11.0	91.8	1.07	3.58	0.09	0.28
Anti-epileptic	Carbamazepine	Carbamazepine-13C6	1.6	92.7	0.27	1.37	0.01	0.03
	Carbamazepine 10,11-epoxide	Carbamazepine-13C6	2.1	89.9	0.53	1.76	-	-
	10,11-Dihydro -10-hydroxycarbamazepine	Carbamazepine-13C6	5.6	93.8	0.99	9.94	0.02	0.25
Calcium-channel blocker	Diltiazem	Carbamazepine-13C6	2.3	93.6	0.27	2.68	-	-
Hypnotic	Temazepam	Temazepam-d5	1.6	97.9	0.18	0.92	0.01	0.04
	Oxazepam	Oxazepam-d5	3.4	94.3	0.20	0.66	-	-
Lifestyle Chemicals	Caffeine	Cotinine-d3	2.8	100.4	121*	581*	-	-
	Cotinine	Cotinine-d3	1.5	98.8	0.27	1.34	0.005	0.02
	1,7-dimethylxanthine[a]	Cotinine-d3	9.9	94.9	560*	2165*	-	-
GUD/ED	Sildenafil	Sildenafil-d8	3.0	99.1	0.01	0.05	0.001	0.003
Anticancer	Capecitabine	Metazachlor-d6	2.8	89.7	0.002	0.01	0.001	0.003

[1]Author for correspondence: B Kasprzyk-Hordern@bath.ac.uk

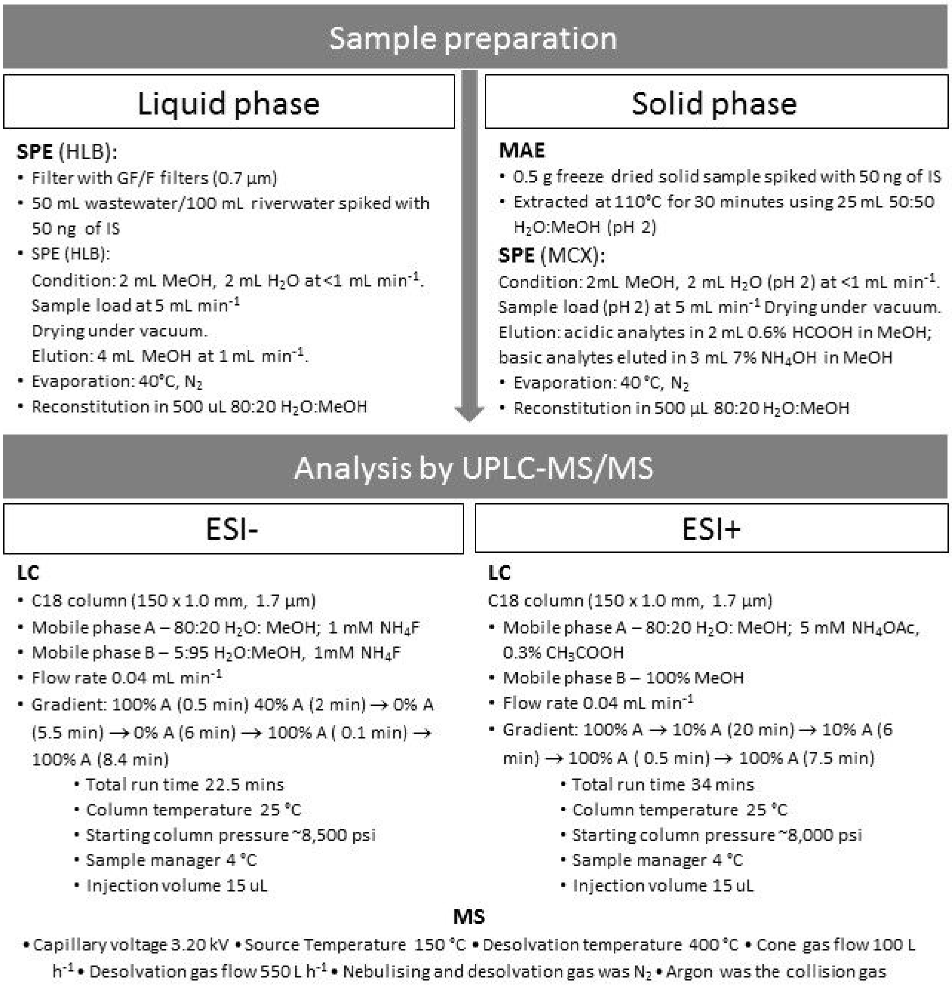

Figure S1. SPE/MAE-UHPLC-QqQ – schematic overview.

Table S2. Daily loads of studied pharmaceuticals in wastewater influent (data supporting Figs 2-5).

**Analyte**		**WWTP A (mg/day)**	**WWTP B (mg/day)**	**WWTP C (mg/day)**	**WWTP D (mg/day)**	**WWTP E (mg/day)**
Propranolol	We	1831.5	2082.4	5050.6	950.4	36728.2
	Thur	1765.4	2274.2	5378.9	1002.2	33869.4
	Fri	1756.0	2121.9	5033.7	937.3	34890.5
	Sat	1670.4	2149.7	4442.1	1078.8	31483.5
	Sun	5829.0	1903.8	4608.2	992.2	34951.6
	Mon	2139.5	2225.2	5341.7	1134.4	33011.1
	Tue	1507.8	2484.6	5722.2	956.8	32638.4
	±SD	±SD	±SD	±SD	±SD	±SD
	We	32.5	10.8	438.4	27.2	262.1
	Thur	43.5	170.6	84.7	29.8	
	Fri	27.1	98.0	735.4	44.9	1823.5
	Sat	31.6	108.7	638.3	26.5	1747.5
	Sun	371.9	18.4	32.7	112.0	101.6
	Mon	182.6	216.6	632.6	17.3	332.1
	Tue	59.6	122.1	167.5	27.6	834.1
Sertraline	We	1333.7	1333.5	3331.0	728.6	29973.4
	Thur	1504.4	2105.8	2936.8	1115.6	31084.7
	Fri	1316.6	1470.6	4804.5	687.3	35662.0
	Sat	1752.7	2019.2	4512.8	2786.0	52154.9
	Sun	2396.8	1209.0	2154.4	835.5	35763.9
	Mon	3289.0	1261.6	4597.6	768.1	43903.3
	Tue	2186.1	3032.2	4854.2	869.8	34979.3
	±SD	±SD	±SD	±SD	±SD	±SD
	We	110.1	54.3	873.5	153.3	1031.4
	Thur	174.3	137.3	156.8	405.2	4294.2
	Fri	60.1	193.2	243.3	52.1	3009.5
	Sat	429.3	483.4	319.1	292.8	2217.0
	Sun	84.5	54.6	130.6	150.1	1066.3
	Mon	1497.4	75.4	237.1	30.5	3477.8
	Tue	279.7	132.6	487.1	64.3	1156.6
Norsertraline	We	897.3	967.4	55716.7	312.8	21593.4
	Thur	877.5	1836.6	111669.5	581.5	26827.6
	Fri	975.2	1089.4	52525.8	248.1	23949.9
	Sat	1410.0	1737.9	128764.8	670.0	50858.2
	Sun	4373.7	862.8	92165.5	529.7	22167.9
	Mon	3342.6	3265.8	38924.8	400.9	55415.9
	Tue	4330.8	2957.9	44811.5	565.0	28287.4
	±SD	±SD	±SD	±SD	±SD	±SD
	We	256.6	37.7	24648.8	167.4	741.3
	Thur	128.1	41.7	20890.8	359.7	7322.0
	Fri	113.2	217.9	3719.6	0.0	5853.1
	Sat	361.8	90.3	31962.9	395.6	3234.5
	Sun	118.8	122.0	37777.5	274.2	2593.2
	Mon	2445.8	62.8	386.0	33.0	16958.4
	Tue	505.2	549.3	5608.0	47.9	1145.3

**Analyte**		**WWTP A (mg/day)**	**WWTP B (mg/day)**	**WWTP C (mg/day)**	**WWTP D (mg/day)**	**WWTP E (mg/day)**
Carbamazepine	We	4100.6	4264.7	6969.3	5773.1	99453.0
	Thur	3707.9	6527.6	7184.6	4101.9	100073.6
	Fri	4192.2	5630.5	8004.8	4010.6	113003.8
	Sat	4041.6	6483.5	7193.6	3779.2	102325.3
	Sun	42946.8	4672.6	6403.8	2249.2	100453.0
	Mon	7345.4	5632.8	9954.6	2477.3	126000.6
	Tue	3627.4	6242.6	8012.4	2551.8	103209.4
	±SD	±SD	±SD	±SD	±SD	±SD
	We	23.28	25.5	49.8	58.4	31.1
	Thur	37.00	59.9	62.2	7.5	0.0
	Fri	27.87	15.3	79.8	62.0	2846.8
	Sat	25.64	43.1	9.5	20.7	71.6
	Sun	43.11	28.3	45.3	13.5	819.5
	Mon	113.83	77.6	124.3	75.4	715.8
	Tue	47.18	65.6	86.7	34.2	616.6
Carbamazepine 10,11-epoxide	We	862.0	1276.5	747.9	478.0	12181.1
	Thur	770.5	1062.1	683.8	516.4	12822.6
	Fri	759.0	946.1	725.5	420.8	9423.9
	Sat	788.2	1222.4	653.0	595.7	15229.8
	Sun	908.9	937.6	618.5	560.9	13529.7
	Mon	766.0	602.5	815.2	671.7	14792.9
	Tue	1095.1	1191.8	874.2	465.0	13809.4
	±SD	±SD	±SD	±SD	±SD	±SD
	We	23.3	25.5	49.8	58.4	31.1
	Thur	37.0	59.9	62.2	7.5	0.0
	Fri	27.9	15.3	79.8	62.0	2846.8
	Sat	25.6	43.0	9.5	20.7	71.6
	Sun	43.1	28.3	45.3	13.5	819.5
	Mon	113.8	77.6	124.3	75.4	715.8
	Tue	47.2	65.6	86.7	34.2	616.6
Capecitabine	We	147.2	97.8	76.7	111.0	2069.0
	Thur	267.1	83.4	40.9	102.1	3589.5
	Fri	142.1	44.5	123.0	408.3	2135.1
	Sat	78.7	36.5	95.9	60.0	4328.2
	Sun	109.2	96.5	217.2	91.9	2824.7
	Mon	137.5	72.5	112.8	67.6	4785.4
	Tue	157.0	0.0	190.7	55.2	2661.6
	±SD	±SD	±SD	±SD	±SD	±SD
	We	2.2	40.9	33.0	0.9	28.3
	Thur	2.6	3.0	10.7	9.7	204.4
	Fri	7.8	16.3	36.1	11.9	205.6
	Sat	25.6	1.5	4.1	4.2	161.5
	Sun	1.4	5.0	40.4	5.8	415.0
	Mon	16.9	0.9	44.5	0.4	1121.3
	Tue	6.6	0.0	9.2	3.9	248.8

Analyte		WWTP A (mg/day)	WWTP B (mg/day)	WWTP C (mg/day)	WWTP D (mg/day)	WWTP E (mg/day)
Sildenafil	We	76.1	64.7	244.4	48.6	2941.6
	Thur	64.9	1391.3	217.3	39.1	2770.7
	Fri	89.3	203.9	306.4	28.9	2974.8
	Sat	162.4	192.7	286.2	48.9	3606.9
	Sun	109.1	166.2	299.3	75.8	3803.8
	Mon	90.8	99.3	384.9	60.2	2813.9
	Tue	108.0	108.7	292.3	45.4	2725.6
	±SD	±SD	±SD	±SD	±SD	±SD
	We	3.3	29.0	40.3	8.1	77.1
	Thur	2.2	66.2	4.4	1.8	503.1
	Fri	0.8	17.3	13.1	2.8	489.7
	Sat	18.7	7.4	32.1	5.6	246.5
	Sun	17.9	34.0	73.2	28.8	139.4
	Mon	20.3	3.4	80.7	2.2	671.8
	Tue	2.4	18.9	50.5	20.9	275.2
Diltiazem	We	1575.0	2584.4	4258.2	1606.7	53299.3
	Thur	1712.4	2556.2	4637.1	5162.3	53344.0
	Fri	1718.1	2551.3	4760.4	4850.7	43937.6
	Sat	1943.9	2856.4	4511.0	1421.9	54969.8
	Sun	3054.4	2363.7	4561.9	1184.3	49407.9
	Mon	2386.1	2992.1	5817.8	1307.9	56089.1
	Tue	1480.1	2190.4	6303.3	1009.8	32597.7
	±SD	±SD	±SD	±SD	±SD	±SD
	We	11.5	95.4	362.6	59.3	1052.9
	Thur	21.5	80.7	215.5	135.9	
	Fri	18.9	144.5	1.6	2.0	1402.8
	Sat	99.9	126.1	47.1	67.6	587.8
	Sun	91.0	108.4	88.2	160.8	1708.9
	Mon	127.8	204.7	145.1	96.2	865.0
	Tue	48.9	6.8	156.8	108.9	181.4
Venlafaxine	We	1859.4	5102.5	8217.1	3133.5	71177.8
	Thur	3164.7	4128.7	6501.9	1450.4	54681.7
	Fri	2131.8	6365.5	6033.8	2351.9	88846.6
	Sat	3098.4	5605.8	5693.3	2675.1	82123.8
	Sun	4263.4	4228.6	5427.3	1715.1	57109.6
	Mon	1896.2	3611.8	11278.2	2085.2	62034.9
	Tue	3409.8	5666.6	9283.1	1245.8	55230.8
	±SD	±SD	±SD	±SD	±SD	±SD
	We	32.5	32.7	422.8	70.9	751.6
	Thur	104.8	20.4	48.7	308.2	-
	Fri	51.8	104.7	41.4	60.4	1699.0
	Sat	147.2	17.7	59.6	76.1	1303.0
	Sun	10.9	63.6	96.2	75.4	3072.4
	Mon	177.5	142.4	56.0	47.1	4251.7
	Tue	65.2	7.7	47.0	1.4	1364.7

Analyte		WWTP A (mg/day)	WWTP B (mg/day)	WWTP C (mg/day)	WWTP D (mg/day)	WWTP E (mg/day)
Desmethylvenlafaxine	We	4830.3	8293.7	11425.2	2914.6	89994.8
	Thur	4877.3	8380.6	11672.5	3526.7	88623.9
	Fri	3985.1	6719.8	11473.0	2859.3	69506.9
	Sat	3742.3	7000.0	10792.6	3289.6	71397.0
	Sun	5336.6	6944.2	10544.8	2663.9	77044.5
	Mon	3242.7	9637.9	14488.0	3454.6	81675.2
	Tue	4323.6	8228.1	13101.6	2123.9	68002.0
	±SD	±SD	±SD	±SD	±SD	±SD
	We	32.5	32.7	422.8	70.9	751.6
	Thur	104.8	20.4	48.7	308.2	-
	Fri	51.8	104.7	41.4	60.4	1699.0
	Sat	147.2	17.7	59.6	76.1	1303.0
	Sun	10.9	63.6	96.2	75.4	3072.4
	Mon	177.5	142.4	56.0	47.1	4251.7
	Tue	65.2	7.7	47.0	1.4	1364.7

Table S3. Daily loads of studied pharmaceuticals (with suspected direct disposal) in wastewater influent and effluent (data supporting Fig 6).

Analyte		Influent (measured) (mg/day)	Effluent (measured) (mg/day)	River downstream (estimated) (mg/day)*
Propranolol (WWTP A)	We	1831.5	1312.1	2296.7
	Thur	1765.4	1162.2	1809.9
	Fri	1756.0	1265.5	2329.4
	Sat	1670.4	1224.2	2006.1
	Sun	5829.0	1217.9	1898.3
	Mon	2139.5	2317.6	3530.9
	Tue	1507.8	1554.2	3048.7
	±SD	±SD	±SD	±SD
	We	32.5	46.0	110.2
	Thur	43.5	47.4	77.7
	Fri	27.1	23.5	65.1
	Sat	31.6	29.6	41.6
	Sun	371.9	17.9	23.6
	Mon	182.6	7.3	82.7
	Tue	59.6	63.6	242.2
Sertraline (WWTP D)	We	728.6	108.4	108.4
	Thur	1115.6	129.0	129.0
	Fri	687.3	140.4	140.4
	Sat	2786.0	175.2	295.7
	Sun	835.5	143.6	264.2
	Mon	768.1	134.4	134.4
	Tue	869.8	110.5	110.5
	±SD	±SD	±SD	±SD
	We	153.3	6.3	6.3
	Thur	405.2	4.1	4.1
	Fri	52.1	13.9	13.9
	Sat	292.8	9.8	64.4
	Sun	150.1	12.7	35.8
	Mon	30.5	5.3	5.3
	Tue	64.3	7.0	7.0
Carbamazepine (WWTP A)	We	4100.6	5078.7	12,166.2
	Thur	3707.9	4716.3	10,624.4
	Fri	4192.2	5090.6	15,193.5
	Sat	4041.6	5359.5	12,498.7
	Sun	42946.8	5573.2	10,213.8
	Mon	7345.4	11705.3	20,924.1
	Tue	3627.4	5511.8	15,548.6
	±SD	±SD	±SD	±SD
	We	23.28	58.7	490.5
	Thur	37.00	22.6	25.1
	Fri	27.87	15.0	379.2
	Sat	25.64	151.8	332.9
	Sun	43.11	52.4	52.4
	Mon	113.83	133.7	245.9
	Tue	47.18	17.8	189.6

Capecitabine (WWTP D)	We	111.0	18.6	18.6
	Thur	102.1	20.4	20.4
	Fri	408.3	94.5	94.5
	Sat	60.0	182.6	182.6
	Sun	91.9	43.2	43.2
	Mon	67.6	11.8	11.8
	Tue	55.2	5.9	5.9
	±SD	±SD	±SD	±SD
	We	0.9	2.6	2.6
	Thur	9.7	2.2	2.2
	Fri	11.9	1.0	1.0
	Sat	4.2	5.5	5.5
	Sun	5.8	5.5	5.5
	Mon	0.4	2.3	2.3
	Tue	3.9	2.4	2.4
Sildenafil (WWTP B)	We	64.7	123.0	123.0
	Thur	1391.3	497.8	642.2
	Fri	203.9	515.4	546.1
	Sat	192.7	322.0	322.0
	Sun	166.2	298.9	298.9
	Mon	99.3	374.7	446.4
	Tue	108.7	274.0	440.7
	±SD	±SD	±SD	±SD
	We	29.0	11.2	11.2
	Thur	66.2	19.9	149.6
	Fri	17.3	30.5	42.7
	Sat	7.4	1.5	1.5
	Sun	34.0	3.2	3.2
	Mon	3.4	9.8	59.8
	Tue	18.9	51.1	165.2
Diltiazem (WWTP D)	We	1606.7	383.1	383.1
	Thur	5162.3	419.6	419.6
	Fri	4850.7	449.2	918.1
	Sat	1421.9	507.0	1024.7
	Sun	1184.3	417.3	417.3
	Mon	1307.9	376.1	376.1
	Tue	1009.8	383.7	383.7
	±SD	±SD	±SD	±SD
	We	59.3	26.6	26.6
	Thur	135.9	5.9	5.9
	Fri	2.0	19.8	19.8
	Sat	67.6	53.2	53.2
	Sun	160.8	1.6	1.6
	Mon	96.2	57.3	57.3
	Tue	108.9	25.8	25.8

* Estimated by adding daily loads in wastewater effluent to loads already present in the river water upstream from a WWTP discharge point.
